# Obstetric Unit Closure Effects on Child Academic Achievement, Infant Health, and Maternal Care

**DOI:** 10.1007/s10995-025-04112-8

**Published:** 2025-06-12

**Authors:** George L. Wehby

**Affiliations:** 1 Department of Health Management and Policy, University of Iowa, 145 N. Riverside Dr., 100 College of Public Health Bldg., Room N232A, Iowa City, IA 52242-2007 USA; 2 Department of Economics, University of Iowa, Iowa City, IA USA; 3 National Bureau of Economic Research, Cambridge, MA USA

**Keywords:** Obstetric unit closures, Perinatal care, Academic achievement

## Abstract

**Objective:**

To examine effects of obstetric unit closures on children’s academic achievement, infant health, and maternal care outcomes.

**Methods:**

This retrospective cohort study employs 1994–2009 birth certificate data from Iowa linked to school test scores for grades 2–11 through 2017–2018. Regressions based on two-way fixed effects (TWFE) and Callaway and Sant’Anna (C&S) difference-in-differences models estimate the effects of maternal residence in counties that had obstetric unit closures within 5 years before birth year. Outcomes are national percentile rankings (NPRs) on math and reading, gestational age, preterm birth, birthweight, low birthweight, prenatal visits, cesarean delivery, and labor induction.

**Results:**

The sample included 2,414,393–2,424,184 child-grade observations and 379,772–381,228 children depending on the outcome. TWFE estimates were − 0.66 (95% CI: − 1.48, 0.15) NPRs for math and − 0.86 (95% CI: − 1.93, 0.21) NPRs for reading. C&S estimates were − 0.88 (95% CI: − 4.48, 2.72) NPRs for math and 0.30 (95% CI: − 4.93, 5.52) NPRs for reading. Closure associations with gestational age were − 0.1 (95% CI: − 0.17, − 0.028) and − 0.09 weeks (95% CI: − 0.28, 0.09) in the TWFE and C&S models, respectively; associations with birthweight were − 33 g (95% CI: − 59, − 7) and − 2 g (95% CI: − 57, 54), respectively. Associations with other infant health and maternal outcomes were small and not statistically significant.

**Conclusions:**

There is overall little evidence that obstetric unit closure within 5 years before birth impact children’s math and reading scores and infant health and maternal care outcomes. Future research can evaluate how closures affect care continuity and access and potential heterogeneity including by maternal health risks and pregnancy complications.

**Supplementary Information:**

The online version contains supplementary material available at 10.1007/s10995-025-04112-8.

## Significance

What’s New

In this study of more than 350,000 children, there was overall no discernable evidence of effects of obstetric unit closures within 5 years before birth on children’s math and reading scores and a range of infant health and maternal care outcomes.

**Supplementary Information:**

The online version contains supplementary material available at 10.1007/s10995-025-04112-8.

## Introduction

The presence of obstetric units in rural areas has been steadily declining over the past 3 decades. Between 1989 and 2019, nearly one fifth of rural counties lost their hospital-based obstetric unit (Fischer et al., 2024). By 2018, less than half of all rural counties had an obstetric unit (Kozhimannil et al., 2020). Driven by multiple factors such as decreasing demand for and revenue from obstetric services and the shortage of specialized workforce, this trend may continue into the foreseeable future.

The reduced access to local obstetric services from these closures has prompted questions and concerns about impacts on maternal and infant health. Closures may disrupt access to prenatal, maternity, and neonatal services and increase travel distances to alternate care sources. Indeed, travel distances for maternity services are farthest for women in rural counties without local obstetric services (Fontenot et al., 2024). However, closure effects on infant and maternal health outcomes depend on multiple factors, namely the extent to which high-risk pregnancies received services from the local obstetric units that closed, and access to and quality of care at alternate care settings. If pregnancies with more maternal and fetal health risks were transferring to regional and higher-volume obstetric centers before closures, then closures may have had less of an effect on the outcomes of these pregnancies. Similarly, closures may have less of an effect on the outcomes of pregnancies with no complications or limited maternal and infant health risk factors. Moreover, differences in quality of care between the closing and new alternative care settings may moderate the effects of care disruptions from closures. For example, if care quality at the alternate care settings is higher than the closing units, this may attenuate the effect of care disruptions (Fischer et al., 2024). For these reasons, the impacts of closures on maternal and infant health outcomes are conceptually ambiguous.

The extant empirical literature has focused on effects of obstetric unit closures on prenatal care and early infant health outcomes such as infant mortality, preterm birth, and low birthweight, and less on long-term child health outcomes. The evidence from the extant studies on these outcomes is somewhat mixed. One study covering closures from 1989 to 2019 and using birth certificate data found declines in deliveries in the county of residence, prenatal visits, and cesarean deliveries and an increase in labor induction, but no discernable effects on maternal and infant health indicators (Fischer et al., 2024). That work also reported some evidence for an increase in deliveries in better resourced and higher quality of care counties. In a working paper that studied closures in rural counties in 2005–2018, Chatterji et al. found an increase in deliveries in urban counties and no discernable effects on infant health with potential heterogeneity by race (Chatterji et al., 2023). Kozhimannil et al. evaluated closures over 2004–2014 in rural counties and reported an increase in deliveries out of hospitals and in hospitals without obstetric units and an increase in preterm birth (Kozhimannil et al., 2018). Lorch et al. studied obstetric unit closures in Philadelphia in 1995–2005 and reported an increase in neonatal mortality (Lorch et al., 2013).

This study extends the literature by examining longer-term effects from obstetric unit closures on children, specifically on their cognitive outcomes measured by school achievement. In addition to academic achievement, the study examines closure effects on infant health and maternal care outcomes as potential pathways. Shocks to infant and maternal health could influence subsequent child health and development in similar directions. If care disruptions from obstetric unit closures meaningfully increase the risk of maternal and fetal health complications and adversely impact infant and early childhood health outcomes, these early life consequences may affect subsequent child health and development including academic outcomes. Indeed, fetal growth and early infant health are associated with children’s academic achievement (Chatterji et al., 2014; Figlio et al., 2014; Wehby, 2022a, 2023). Moreover, disruptions in maternal and perinatal care services may affect other dimensions of early infant health such as the need for and speed of admission to neonatal intensive care or other early interventions that may also have long term effects on children’s development. Children’s academic achievement is related to their physical and mental health conditions (Eide et al., 2010; Guarnizo-Herreno et al., 2019), which in turn are related to fetal growth and early infant health.

The study focuses on obstetric unit closures in the state of Iowa between 2000 and 2005. Iowa had 41 obstetric unit closures between 2000 and 2021 in 39 of its 99 counties (Rouse et al., 2022). Considering this relatively large number of closures and that Iowa is among the states with the largest proportions of populations residing in rural areas (36.8% in 2020 versus 20% of the total US population; US Census Bureau, 2023, 2024), understanding the effects of these closures in Iowa is important both for the state and other states with similar closure trends and rates of residence in rural areas. Because the study examines a long-term outcome and because of the availability of achievement data (described below), only the earlier closures between 2000 and 2005 are included. Closures are expected to have immediate effects on maternal and infant health and subsequently on the academic achievement of children born soon after closures. Closures may also affect children born many years later in the areas that experienced a closure. However, including cohorts born many years after a closure increases the likelihood of confounding time trends and other unobserved events. Moreover, adjustment to finding new sources of care is more likely after longer time from closures. Therefore, the study design (described below) focuses on outcome changes over five birth year cohorts following closure (i.e. 5 birth years including the closure year) in the counties experiencing a closure.

## Methods

### Data and Sample

The study data come from linking restricted-access birth certificates in Iowa and standardized school tests. The study focuses on tests in grades 2 through 11 because there is much less testing in K-1 and 12 grades. Details for the data linkage are described elsewhere (Wehby, 2022a, 2022b, 2023). The school test data were available to the study through school year 2017–2018. Since the study includes tests beginning in grade 2, the study focuses on births through 2009 as most children born in subsequent years would not have school test data in this period.

Data on hospital-based obstetric unit closures from 2000 onward are from the Iowa Department of Health and Human Services (Iowa HHS, formerly the Iowa Department of Public Health). The data were compiled by staff at the Bureau of Family Health from hospital reports on closures to the bureau’s Newborn Metabolic Screening and the Early Hearing Detection and Intervention Programs. Bureau staff followed up with the hospitals’ nursing leaders to verify closure dates when possible (Rusty S., email communication, January 12, 2023). There were no data from this source on closures before 2000. Additional data on availability of obstetric units by county since 2000 come from recent Iowa HHS reports and other data received from Bureau of Family Health staff about obstetric unit availability in 2023 (Iowa HHS, 2023; Division of Health Promotion & Chronic Disease Prevention, 2022; Rusty S., email communication, February 14, 2023).

As mentioned above, the study focuses on closures between 2000 and 2005 and on the five birth years post closures to assess effects on children most likely to be affected and minimize the threat of contemporaneous confounders over longer time periods following closures. The design also focuses on five birth years before the year preceding closure, so 1994 is the first birth year included in the sample. Therefore, the sample includes births from the 6 years before closure and 5 years after closure (including the closure year) for counties that experienced an obstetric unit closure.

Two counties (Adams and Mitchell) had closures in 2006 and 2008 and were excluded from the sample because there were no school test score data over all five birth years following closures in these counties (because the last year of school test data is 2017–2018 as noted above). Between 2000 and 2005, there were 12 obstetric unit closures in 12 counties. One of those closures was in a county (Dubuque) which had two other level II obstetric units, so that county was excluded as the closure would likely have less of an impact given the presence of other units. Three closures were in counties that had one other level I obstetric unit (Fayette, Hardin, and Page), and so were excluded from the main sample but included in a sensitivity analysis. Therefore, the main analytical sample focuses on 8 counties that lost their only obstetric unit in 2000–2005. Moreover, 25 counties that did not have any obstetric unit anytime in 2000–2009 were excluded from the study as some of these counties could have experienced earlier closures (not available in the closure data referenced above) and some may have experienced spillover effects from closures during the study period. Therefore, the sample includes counties that experienced closures between 2000 and 2005 (except for the exclusions noted above) and counties that always had at least one obstetric unit throughout the study birth period.

The main sample is further limited to singleton births given the higher risk of adverse birth outcomes with multiple births. This higher risk identified early on in pregnancy may result in early referral to regional obstetric units. However, we consider an alternate sample that includes multiple births in a sensitivity analysis.

### Outcome Measures

The outcomes are math and reading scores in national percentile rankings (NPRs) from standardized school tests (Iowa Tests of Basic Skills, Iowa Tests of Educational Development, and Iowa Assessments) administered in public and private schools (Hoover et al., 2003). These scores have been associated with birth outcomes and other infant health indicators relevant for subsequent children’s health and development and with family insurance eligibility expansions (Collett et al., 2014; Gallagher et al., 2017; Wehby et al., 2015; Wehby, 2022a, 2022b, 2022c, 2023). Other outcomes from the birth certificates include birthweight (in grams), low birthweight (< 2500 g), gestational age (weeks), preterm birth (< 37 weeks), number of prenatal visits, cesarean delivery, and labor induction.

### Statistical Analysis

This is a retrospective cohort study with variation in closure status and timing across counties. Because of the staggered timing of closures across counties and the child-level data with repeated outcomes over time for each child (grade), the analysis begins with a two-way fixed effect (TWFE) regression model that includes a time-varying county-level indicator for closures and county and year fixed effects along with a range of child and school test specific covariates as follows:

$$TestScore_{icbt} = \beta .Closure_{cb} + \,\,C_{c} + B_{b} + T_{t} + S_{icbt} + X_{icb} + e_{icbt} .$$

In model (1), *TestScore* is the math or reading test score in year *t* for child *i* born in year *b* whose mother resided in county *c* during pregnancy. Closure is measured at the county level. Because of the relatively low population density in several counties including those where closures happened, the county captures more fully the geographic area served by local obstetric units in rural areas than smaller units such as the zip-code. Accordingly, *Closure* is a 0/1 indicator equal to 1 for births in the year of or after the obstetric unit closure in a given county. In a sensitivity analysis, we switch the closure indicator to 1 beginning in the year before closure year to consider the possibility changes in local obstetric care availability and access before closure. *C*, *B*, and *T* include fixed effects (0/1 indicators) for county of maternal residence during pregnancy, child’s birth year, and test year, respectively. *S* includes fixed effects for grade and semester of testing. *X* includes indicators for the child’s sex and maternal age, marital status, education, race/ethnicity, and number of prior live births at the child’s birth. A similar model is estimated for infant and maternal outcomes based on the sample of unique children without the test specific covariates (*S*).

The TWFE model is flexible in accommodating the time-varying measurement of closures, the large sample of child-grade observations, and multiple child-level covariates. However, that model assumes that the closure effect does not change over time after closure. In other words, it assumes that the closure effect is the same in years 1 through 5 following closure (since 5 years post-closure are included in this study). If that assumption is incorrect, the TWFE estimates would be biased since counties experiencing earlier closures serve as controls for counties experiencing later closures (Goodman-Bacon, 2021). Even though closure effects may not necessarily increase over time (by birth year after closure), such time effect heterogeneity cannot be ruled out. For this reason, another difference-in-differences type model that also incorporates staggered timing innovated by Callaway and Sant’Anna (henceforth referred to as C&S) is employed that is robust to this issue (Callaway & Sant’Anna, 2021). Specifically, the model involves a series of difference-in-differences comparisons for each “cohort” of counties that experienced a closure based on year of closure, where each cohort is compared to counties that never experienced a closure. From these comparisons, the model aggregates the cohort-specific effects into an average treatment effect. The model also generates event-study estimates for how effects change over time since closure but also pre-trends for how outcomes changed over time before closure in order to test the validity of this research design. We report estimates from both the TWFE and the C&S models to evaluate the sensitivity of the results to the potential heterogeneity in treatment effects over time.

To implement the C&S model, the outcome data are collapsed to the county and birth year level. The C&S model allows for including covariates. However, the birth certificate sociodemographic covariates are more appropriate for explaining the individual-level variation in the outcomes based on the child-grade (for math and reading scores) or child-level (for infant and maternal outcomes) data than the data collapsed over counties and years. Therefore, four county-level socioeconomic covariates are included in the C&S model: percent of families below poverty line, median family income, percent of population below high school education or less, and percent unemployed. These covariates are measured in 1990 before the beginning of the study period from the Surveillance, Epidemiology, and End Results (SEER) County Attributes files (SEER, 2024). The inclusion of these covariates considers the possibility of outcomes evolving over time differently across counties based on these “baseline” indicators but does not rule out that closures might subsequently lead to disruptions in local labor markets and effects on income in the counties experiencing closures which could affect the evaluated outcomes. In other words, any effect from closures on local economic conditions is still captured in the effect estimate.

The C&S difference-in-differences model is estimated using the doubly robust estimator with stabilized inverse probability weighting. Confidence intervals (CIs) are estimated based on bootstrapped standard errors clustered at the county level. Effect estimates are considered statistically significant based on a 5% type-1 error two-sided test. Data analysis was performed in 2023–2025 using STATA version 15.1 (StataCorp, 2017).

## Results

### Sample Description

The primary analytical sample includes births in 71 counties, 8 of which had a closure during the study period. In an alternate sample, 3 more counties that had closures but also another level I obstetric unit are added for a total of 11 counties that experienced closures. Appendix Table 1 online includes the counties in the sample and closure status and year.

Table 1 summarizes the study outcomes and sociodemographic characteristics of the primary analytical sample. The primary analytical sample included 2,414,393 child-grade observations for math scores and 2,424,184 child-grade observations for reading scores, and 379,772 to 381,228 children for the infant health and maternal outcomes (depending on the outcome). Mean math and reading scores were 62 and 61 NPRs, respectively. Preterm and low birthweight rates were 6.7% and 4.3%, respectively. On average, mothers had about 12 prenatal visits, 23% had a cesarean delivery, and 24% had a labor induction.

**Table 1:** Analytical sample description

School test scores	
Math score, Mean (SD)	62.18 (26.77)
Reading comprehension score, Mean (SD)	61.30 (27.12)
Infant Health Outcomes	
Gestational Age (Weeks), Mean (SD)	38.96 (1.75)
Preterm birth (%)	
Yes	6.7
No	93.3
Birthweight (grams), Mean (SD)	3424 (537)
Low birthweight (%)	
Yes	4.3
No	95.7
Maternal Outcomes	
Prenatal visits, Mean (SD)	11.9 (3.2)
Cesarean delivery (%)	
Yes	23.0
No	77.0
Labor Induction (%)	
Yes	24.4
No	75.6
Demographic, Socioeconomic, and Infant Health Variables	
Child’s sex (%)	
Female	49.1
Male	50.9
Number of prior live births (%)	
0	39.6
1	34.4
2	17.3
3	6.0
4 or more	2.7
Maternal age at child’s birth (years), (%)	
< 20	9.2
20–24	25.1
25–29	32.0
≥ 30	33.8
Maternal race (%)	
White	94.6
Other race	5.4
Maternal Hispanic ethnicity (%)	
Yes	4.8
No	95.2
Maternal marital status at child’s birth (%)	
Married	72.8
Not married	27.2
Maternal education at child’s birth (%)	
Less than high school	12.7
High school	29.5
Some college	30.2
College graduate	27.6
Child has congenital anomalies (%)	
Yes	0.2
No	99.8

The summary statistics for the outcomes are based on the regression samples (child-grade observations for math and reading scores and unique children for infant and maternal outcomes). The summary statistics for the demographic, socioeconomic, and infant health covariates are based on the regression sample of child-grade observations for math scores as the outcome. The sample sizes are as shown in Table 2 below

### TWFE Estimates

Table 2 reports the TWFE estimates of obstetric unit closure effects on math and reading scores. The effect estimates for math and reading were − 0.66 (95% CI: − 1.48, 0.15) and − 0.86 (95% CI: − 1.93, 0.21) NPRs, respectively. The effect estimates for the infant health and maternal outcomes are also shown in Table 1. Estimates for most outcomes are small and not statistically significant. Closures are associated with lower gestational age (− 0.1 weeks, 95% CI: − 0.17, − 0.028) and birthweight (− 33 g, 95% CI: − 59, − 7).

**Table 2:** TWFE estimates of obstetric unit closure effects on school test scores and infant and maternal outcomes

Outcome	Effect Estimate	95% CI	N
School tests^a^			
Math scores	− 0.66	− 1.48, 0.15	2,414,393
Reading scores	− 0.86	− 1.93, 0.21	2,424,184
Infant outcomes^b^			
Gestational age (weeks)	− 0.099	− 0.17, − 0.028	380,319
Preterm birth	0.0044	− 0.0037, 0.012	380,319
Birthweight (grams)	− 33.1	− 59.2, − 7.09	381,228
Low birthweight	0.0072	− 0.0019, 0.016	381,228
Maternal outcomes^b^			
Number of prenatal visits	0.0034	− 0.21, 0.22	379,772
Cesarean delivery	− 0.0091	− 0.022, 0.0037	381,053
Labor induction	− 0.0038	− 0.029, 0.022	380,872

*TWFE* Two-way fixed effects

^a^Sample is based on child-grade observations

^b^Sample is based on unique children

Appendix Table 2 online reports the sensitivity analysis results adding the three counties that had closures but also another level I obstetric unit. Results are generally similar with smaller estimates for test scores and birthweight (with the birthweight estimate switching signs and no longer being statistically significant). Appendix Table 3 online reports the results adding multiple births. Estimates are comparable to the main sample results. Lastly, Appendix Table 4 online reports the estimates from a model that allows for closure effects to occur 1 year before closures (i.e. switches the year before closure to a closure year). In that sensitivity analysis, results are overall comparable, although the decline in reading is more pronounced and statistically significant (− 1.44 NPRs; 95% CI: − 2.70, 0.19). 

### C&S Estimates

Table 3 reports the aggregated C&S difference-in-difference estimates of closure effects on math and reading scores and on the infant and maternal outcomes for the main sample. The estimates are − 0.88 (95% CI: − 4.48, 2.72) NPRs for math and 0.30 (95% CI: − 4.93, 5.52) NPRs for reading. Effect estimates for the infant and maternal outcomes are small and not statistically significant. The C&S point estimate for gestational age decline is close to the TWFE estimate but not statistically significant (− 0.09, 95% CI: − 0.28, 0.09 weeks).

**Table 3:** C&S differences-in-differences aggregated estimates of obstetric unit closure effects on school test scores and infant and maternal outcomes

Outcome	Effect estimate	95% CI
School tests		
Math scores	− 0.88	− 4.48, 2.72
Reading scores	0.30	− 4.93, 5.52
Infant outcomes		
Gestational age (weeks)	− 0.092	− 0.276, 0.092
Preterm birth	0.009	− 0.015, 0.034
Birthweight (grams)	− 1.7	− 56.8, 53.5
Low birthweight	0.004	− 0.012, 0.020
Maternal outcomes		
Number of prenatal visits	0.174	− 0.247, 0.595
Cesarean delivery	− 0.005	− 0.034, 0.024
Labor induction	− 0.008	− 0.056, 0.0401

Analytical sample includes outcome means for county-by-year observations. *C&S* Callaway & Sant’Anna (2021)

The C&S results from the sensitivity analysis adding the three counties that had closures but also another level I obstetric unit are mostly similar to the main results (Appendix Table 5 online). The C&S results are also overall comparable when adding multiple births (Appendix Table 6 online). Lastly, when allowing for closure effects 1 year before closure, the aggregated C&S estimates are more pronounced for math and reading scores with a statistically significant decline in math scores (− 1.62 NPRs; 95% CI:− 2.87,− 0.38, Appendix Table 7 online).

**Fig. 1:**
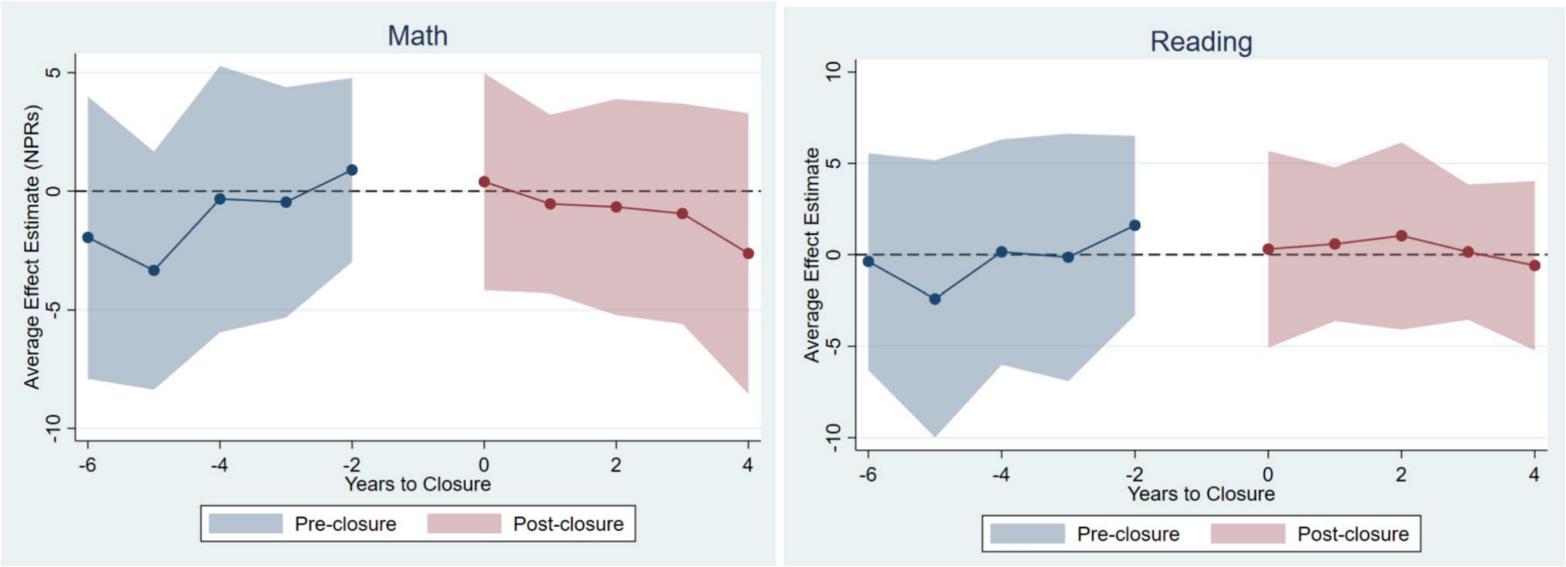
C&S Difference-in-differences event-study estimates of OB unit closure effects on math and reading scores. The dots represent the difference-in-difference event study estimates. The shaded area represents 95% confidence intervals for the estimates. Analytical sample includes outcome means for county-by-year observations. *C&S* Callaway & Sant’Anna (2021). The reference year is the year before closure

Figure 1 shows the C&S event-study estimates of closure effects on math and reading. Most post-closure estimates are close to the null and none is statistically significant. Similarly, none of the pre-closure trend estimates is statistically significant and there is no evidence of systematic pre-trend differences in test scores before closure.

**Fig. 2:**
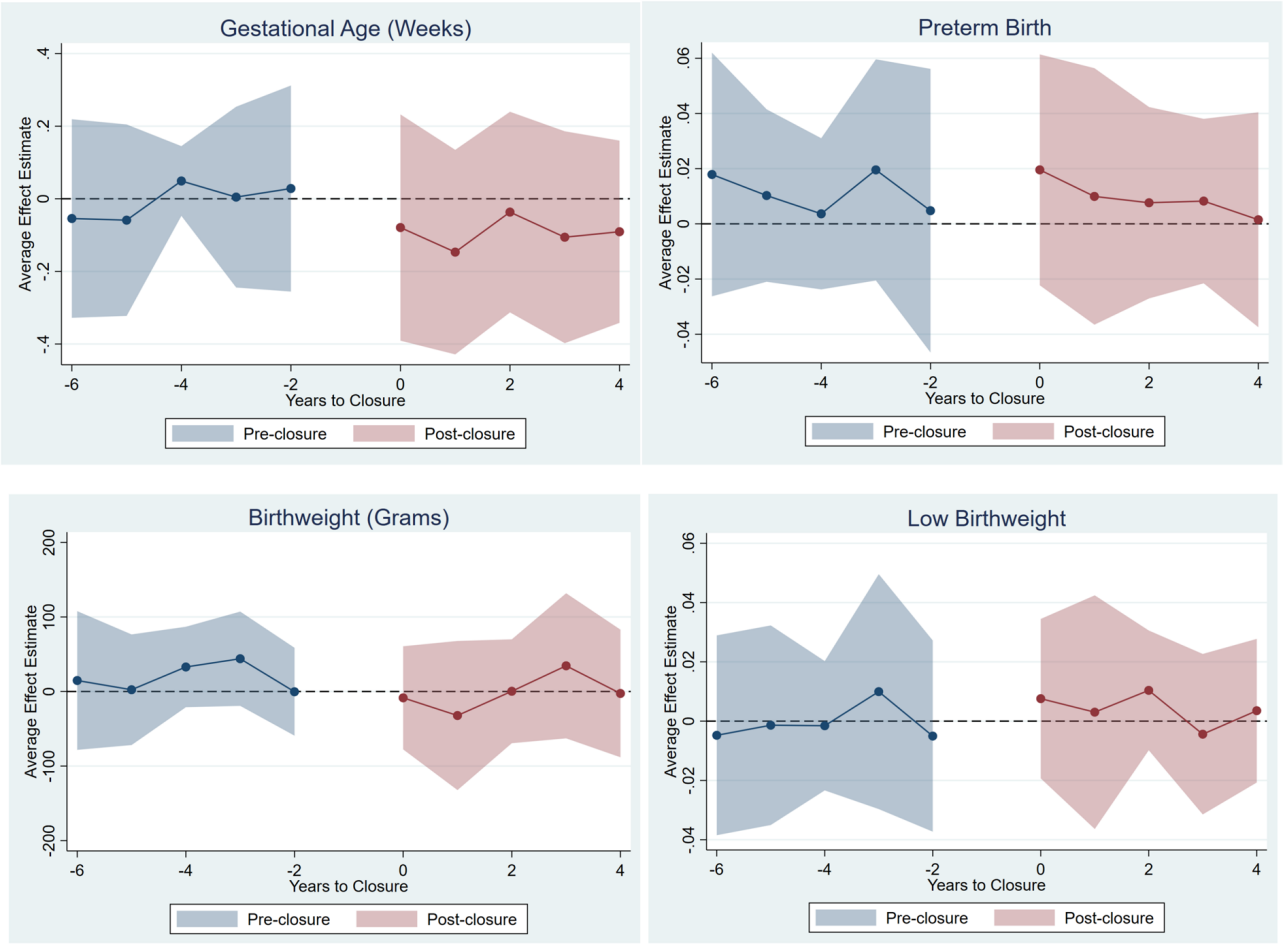
C&S difference-in-differences event-study estimates of obstetric unit closure effects on infant outcomes. The dots represent the difference-in-difference event study estimates. The shaded area represents 95% confidence intervals for the estimates. The point estimates are in weeks for gestational age, grams for birthweight, and 0–1 likelihood change for preterm birth and low birthweight. Analytical sample includes outcome means for county-by-year observations. *C&S* Callaway & Sant’Anna (2021). The reference year is the year before closure

**Fig. 3:**
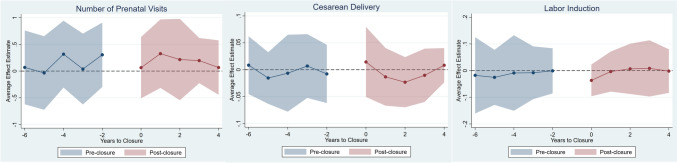
C&S Difference-in-Differences Event-Study Estimates of Obstetric Unit Closure Effects on Maternal Outcomes. The dots represent the difference-in-difference event study estimates. The shaded area represents 95% confidence intervals for the estimates. The point estimates are in 0–1 likelihood changes for the outcomes. Analytical sample includes outcome means for county-by-year observations. *C&S* Callaway & Sant’Anna (2021). The reference year is the year before closure

Figures 2 and 3 show the C&S event-study estimates for the infant and maternal outcomes, respectively. Overall, there is no evidence of closure effects on these outcomes (none of the post-closure estimates are statistically significant). Also, there are no systematic pre-trends.

Appendix Figure 1 online shows the C&S event-study estimates for math and reading using 2 years before closure as the reference year to check if there are potential effects in the year before closure. There are no significant effects on either outcome in the year before closure relative to the prior year or in any other year following closure, except for a decline in math in the fifth year following closure (relative to 2 years before closure).

Appendix Figures 2 and 3 online show the C&S event-study estimates using 2 years before closure as the reference year for the infant and maternal outcomes, respectively. There are no significant effects of closure in the year before closure or in any year following closure.

## Discussion

This study evaluates the effects of obstetric unit closures on children’s academic achievement measured by standardized school test scores on math and reading and on infant health and maternal care outcomes. Overall, there is little evidence for effects on the academic achievement of children born during the same years of closure or within 5 years following closures. When allowing for the closure effect to begin 1 year before closure in case of reduced care resources and availability before closure, there is some decline in the school test scores when aggregating the effects across all 6 years from 1 year before closure to 5 years after closure. The event-study results, however, show no effects in the year before closure and the only statistically significant estimate of closure effects is a decline in math scores 5 years post closure. Not observing significant effects for births sooner after closures suggests that this decline is potentially spurious. Moreover, there are no discernable effects on infant health and maternal care outcomes. Although some estimates suggest declines in gestational age and birthweight, the estimated decline in gestational age up to 0.1 weeks is small and not clinically significant. The birthweight decline suggested in some estimates, while potentially more meaningful in magnitude (22–35 g) is not observed in all models and some estimates suggest no effects on birthweight.

This study is among the first to examine the effects of obstetric unit closures on children’s achievement and long-term development outcomes beyond infant health. Because of the lack of prior research on long-term effects of closures on child health and development, the study findings on academic achievement cannot be directly compared to prior studies that examined a similar outcome or other long-term developmental outcomes. However, the findings from this study on infant health and maternal care outcomes are generally within range of results from recent studies using national data and accounting for time-invariant differences between the areas of closure and non-closure and time trends (Chatterji et al., 2023; Fischer et al., 2024). Even though the findings from this study are specific to one state, they are broadly consistent with the findings of those studies using national data suggesting no discernable meaningful effects of these closures on infant health and maternal care outcomes (Chatterji et al., 2023; Fischer et al., 2024).

The study finds no effects on the evaluated maternal care measures which are prenatal visits, cesarean delivery, and labor induction. This finding suggests that individuals who would have sought prenatal and obstetric services at the closing units may have had access to alternate sources of care with comparable patterns of obstetric care delivery and quality as captured by these outcomes and that facilitate continuity of prenatal and obstetric care. A potential explanation for the lack of effects on these maternal care measures and the evaluated infant health and academic outcomes is that high-risk pregnancies were already being referred to regional and higher-level care obstetric centers earlier in pregnancy and so were not as much affected by disruptions from local obstetric unit closures. Similarly, care disruptions from closures may have had limited impacts on the outcomes of pregnancies with lower risks. Importantly however, the study provides “on-average” estimates, and it is possible that there is a proportion of the sample who experience care disruption and impacts on outcomes not captured in these estimates.

The study has some limitations. The study evaluates a relatively short period of obstetric unit closures (from 2000 to 2005) due to the currently available duration of academic achievement data and lack of data on earlier obstetric unit closures. As mentioned above, 41 closures have occurred in Iowa between 2000 and 2021. However, because of investigating a long-term outcome later in childhood, it is not currently possible to investigate the effects of all those closures on academic achievement. Investigating more closures in the future can enhance power and generalizability. Also, the study is specific to one state which may limit the generalizability of findings. However, findings on infant health and maternal care outcomes are generally within range of previous studies with broader samples as noted above. Another limitation is that some of the C&S estimates are not precisely estimated with relatively wide confidence intervals because of the small number of closures, averaging outcomes over counties, and omitting individual-level covariates from that model. Those estimates should be interpretated considering this caveat. Comparatively, most C&S aggregated point estimates are within range of or close to the TWFE confidential intervals. There is also potential measurement error in recording labor induction on birth certificates. However, this measurement error is unlikely to differ between counties that experience closure and those that do not. In that case, such error would not bias the effect estimate but increases the standard error (making the effect estimate on labor induction less precise). Lastly, it is not possible to rule out residual confounding despite accounting for time trends and time-invariant differences between counties. Potential confounders such as local economic disruptions or demographic trends that may contribute to obstetric unit closures would likely bias the estimates towards negative effects on outcomes. Observing overall no discernable evidence for negative effects suggests little such bias in the results.

The study offers important future research directions that would also address some of the above-discussed limitations. It is important to investigate the long-term effects of obstetric unit closures using alternate datasets that cover more closures and geographies. Such datasets will increase statistical power but also include broader geographic and socioeconomic representation to study these effects. Future research can also evaluate potential heterogeneity in closure effects by distance to and quality of alternate sources of care, socioeconomic factors that impact access such as income and insurance coverage, and prenatal health risks such as maternal chronic conditions. Calculating traveling distance between residence and settings of prenatal, delivery, and postpartum care using finer geographic measures and units can capture impacts more precisely. Also, understanding potential heterogeneity in closure effects and care referral patterns by maternal health risks and pregnancy complications can explain the apparent lack of the “on-average” effects as observed in this study. Moreover, understanding other domains of care access and quality such as waiting times for scheduling appointments and receiving care from the same provider are important. Lastly, obstetric unit closures may affect other long-term health and development outcomes such as mental and behavioral health. Examining such outcomes is critical to fully understand closure effects on children and their families. Such research would benefit from innovative linkages of birth record data and other datasets that provide granular measures of maternal health risks and children’s healthcare utilization and health status such as insurance claims, hospital discharge data, and electronic health records.

## Conclusion

In one of the first studies to evaluate long-term effects of obstetric unit closures on children’s development, this study finds overall little evidence that obstetric unit closures impact the academic achievement of children born within 5 years from closures. Similarly, there is overall no discernable evidence that such closures affect a range of infant health and maternal care outcomes. Additional research can evaluate closure effects on care disruptions and referrals considering travel distances and care continuity and potential heterogeneity by maternal health risks and pregnancy complications.

## Supplementary Information

Below is the link to the electronic supplementary material.

Supplementary file1 (DOCX 986 KB)

## Data Availability

The data of this study comes from data sources governed by data use agreements (with the Iowa Department of Health and Human Services (IDHHS), formerly the Iowa Department of Public Health and with the Iowa Testing Programs (ITP)) that prohibit the researchers from sharing these datasets with others. Researchers interested in accessing this data should contact the IDHHS and ITP for data access.

## Abbreviations

TWFE: Two-way fixed effects
NPRs: National percentile rankings
C&S: Callaway and Sant’Anna
IDHHS: Iowa Department of Health and Human Resources
SEER: Surveillance, Epidemiology, and End Results
CI: Confidence interval

## Code Availability

Statistical code for analyses (in STATA) is available upon request.
