# Supplementary material for: Obstetric Unit Closure Effects on Child Academic Achievement, Infant Health, and Maternal Care
Source: Matern Child Health J. 2025 Jun 12;29(6):825–34. doi: 10.1007/s10995-025-04112-8 (PMC12206179; doi:10.1007/s10995-025-04112-8)
Supplement: Supplementary file 1 — Supplementary file1 (DOCX 986 KB) [file 10995_2025_4112_MOESM1_ESM.docx]

**Appendix**

**Appendix Table 1: Counties Included in Analytical Sample**

| Allamakee | Fayette^2,4^ | Montgomery |
| --- | --- | --- |
| Appanoose | Floyd | Muscatine |
| Audubon^1^ | Franklin | O'brien |
| Black Hawk | Fremont^3^ | Osceola |
| Boone | Greene | Page*^1,4^* |
| Bremer | Hamilton | Palo Alto |
| Buchanan^1^ | Hardin^3,4^ | Plymouth |
| Buena Vista | Henry | Polk |
| Calhoun | Howard | Pottawattamie |
| Carroll | Humboldt^2^ | Poweshiek |
| Cass | Ida | Sac^1^ |
| Cerro Gordo | Jackson | Scott |
| Cherokee | Jasper | Shelby |
| Chickasaw | Jefferson^3^ | Sioux |
| Clay | Johnson | Story |
| Clayton | Jones^3^ | Union |
| Clinton | Kossuth | Van Buren |
| Crawford | Lee | Wapello |
| Dallas | Linn | Washington |
| Davis | Lucas | Wayne |
| Decatur^2^ | Lyon | Webster |
| Delaware | Mahaska | Winneshiek |
| Des Moines | Marion | Woodbury |
| Dickinson | Marshall | Wright |
| Emmet | Monona |  |

Notes: *^1^* Counties experienced an obstetric unit closure in 2005. ^2^ Counties experienced an obstetric unit closure in 2004. ^3^ Counties experienced an obstetric unit closure in 2000. ^4^ Counties had another level I obstetric unit.

**Appendix Table 2. TWFE Estimates of Obstetric Unit Closure Effects on School Test Scores and Infant and Maternal Outcomes Adding Three Counties with Closure but also Another Obstetric Unit**

| Outcome | Effect Estimate | 95% CI | N |
| --- | --- | --- | --- |
| *School Tests^a^* |  |  |  |
| Math scores | -0.13 | -0.98, 0.73 | 2447938 |
| Reading scores | -0.42 | -1.28, 0.4 | 2457630 |
| *Infant Outcomes^b^* |  |  |  |
| Gestational age (weeks) | -0.10 | -0.16, -0.039 | 385354 |
| Preterm birth | 0.0050 | -0.00052, 0.011 | 385354 |
| Birthweight (grams) | -22.7 | -44.2, -1.23 | 386271 |
| Low birthweight | 0.0061 | -0.00020, 0.012 | 386271 |
| *Maternal Outcomes^b^* |  |  |  |
| Number of prenatal visits | 0.078 | -0.36, 0.52 | 379655 |
| Cesarean delivery | -0.0032 | -0.018, 0.012 | 386087 |
| Labor induction | -0.0067 | -0.035, 0.021 | 385899 |

Notes: *^a^* Sample is based on child-grade observations; *^b^* sample is based on unique children. TWFE=Two-way fixed effects.

**Appendix Table 3. TWFE Estimates of Obstetric Unit Closure Effects on School Test Scores and Infant and Maternal Outcomes Adding Multiple Births**

| Outcome | Effect Estimate | 95% CI | N |
| --- | --- | --- | --- |
| *School Tests^a^* |  |  |  |
| Math scores | -0.80 | -1.65,0.059 | 2489147 |
| Reading scores | -0.95 | -1.96,0.070 | 2499208 |
| *Infant Outcomes^b^* |  |  |  |
| Gestational age (weeks) | -0.071 | -0.14,-0.00090 | 392397 |
| Preterm birth | 0.0029 | -0.0065,0.012 | 392397 |
| Birth weight (grams) | -26.9 | -50.4,-3.30 | 393308 |
| Low birth weight | 0.0040 | -0.0053,0.013 | 393308 |
| *Maternal Outcomes^b^* |  |  |  |
| Number of prenatal visits | 0.024 | -0.19,0.23 | 386433 |
| Cesarean delivery | -0.010 | -0.024,0.0041 | 393133 |
| Labor induction | -0.0022 | -0.028,0.023 | 392905 |

Notes: *^a^* Sample is based on child-grade observations; *^b^* sample is based on unique children. TWFE=Two-way fixed effects.

**Appendix Table 4. TWFE Estimates of Obstetric Unit Closure Effects on School Test Scores and Infant and Maternal Outcomes Allowing for Closure Effect One Year before Closure**

| Outcome | Effect Estimate | 95% CI | N |
| --- | --- | --- | --- |
| *School Tests^a^* |  |  |  |
| Math scores | -0.89 | -1.80,0.012 | 2414393 |
| Reading scores | -1.44 | -2.70,-0.19 | 2424184 |
| *Infant Outcomes^b^* |  |  |  |
| Gestational age (weeks) | -0.098 | -0.15,-0.049 | 380319 |
| Preterm birth | 0.0041 | -0.0053,0.013 | 380319 |
| Birth weight (grams) | -35.1 | -51.8,-18.3 | 381228 |
| Low birth weight | 0.0070 | -0.0026,0.017 | 381228 |
| *Maternal Outcomes^b^* |  |  |  |
| Number of prenatal visits | -0.063 | -0.37,0.25 | 374773 |
| Cesarean delivery | -0.0078 | -0.026,0.011 | 381053 |
| Labor induction | -0.012 | -0.042,0.017 | 380872 |

Notes: *^a^* Sample is based on child-grade observations; *^b^* sample is based on unique children. TWFE=Two-way fixed effects.

**Appendix Table 5. C&S Difference-in-Differences Aggregated Estimates of Obstetric Unit Closure Effects on School Test Scores and Infant and Maternal Outcomes Adding Three Counties with Closure but also Another Level I Obstetric Unit**

| Outcome | Effect Estimate | 95% CI |
| --- | --- | --- |
| *School Tests* |  |  |
| Math scores | -0.50 | -4.08, 3.09 |
| Reading scores | 0.32 | -2.80, 3.43 |
| *Infant Outcomes* |  |  |
| Gestational age (weeks) | -0.071 | -0.205, 0.063 |
| Preterm birth | 0.009 | -0.010,0.027 |
| Birthweight (grams) | 3.1 | -38.6, 44.9 |
| Low birthweight | 0.002 | -0.010, 0.014 |
| *Maternal Outcomes* |  |  |
| Number of prenatal visits | 0.31 | -0.14, 0.756 |
| Cesarean delivery | 0.004 | -0.021, 0.029 |
| Labor induction | -0.008 | -0.056, 0.041 |

Notes: Analytical sample includes outcome means for county-by-year observations. C&S=Callaway & Sant’Anna (2021).

**Appendix Table 6. C&S Differences-in-Differences Aggregated Estimates of Obstetric Unit Closure Effects on School Test Scores and Infant and Maternal Outcomes Adding Multiple Births**

| Outcome | Effect Estimate | 95% CI |
| --- | --- | --- |
| *School Tests* |  |  |
| Math scores | -1.28 | -4.77,2.21 |
| Reading scores | 0.11 | -3.08,3.30 |
| *Infant Outcomes* |  |  |
| Gestational age (weeks) | -0.06 | -0.26,0.15 |
| Preterm birth | 0.004 | -.027,0.035 |
| Birth weight (grams) | 10.5 | -49.4,70.4 |
| Low birth weight | 0.002 | -0.016,0.020 |
| *Maternal Outcomes* |  |  |
| Number of prenatal visits | 0.195 | -0.227,0.617 |
| Cesarean delivery | -0.011 | -0.046,0.025 |
| Labor induction | -0.002 | -0.048,0.043 |

Notes: Analytical sample includes outcome means for county-by-year observations. C&S=Callaway & Sant’Anna (2021)

**Appendix Table 7. C&S Differences-in-Differences Aggregated Estimates of Obstetric Unit Closure Effects on School Test Scores and Infant and Maternal Outcomes Allowing for Closure Effect One Year before Closure**

| Outcome | Effect Estimate | 95% CI |
| --- | --- | --- |
| *School Tests* |  |  |
| Math scores | -1.62 | -2.87,-0.38 |
| Reading scores | -1.4 | -3.69, 0.88 |
| *Infant Outcomes* |  |  |
| Gestational age (weeks) | -0.10 | -0.21,0.007 |
| Preterm birth | 0.002 | -0.019,0.023 |
| Birth weight (grams) | -2.2 | -47.7,43.2 |
| Low birth weight | 0.008 | -0.008,0.024 |
| *Maternal Outcomes* |  |  |
| Number of prenatal visits | -0.16 | -0.42,0.12 |
| Cesarean delivery | 0.004 | -0.036,0.044 |
| Labor induction | -0.003 | -0.031,0.024 |

Notes: Analytical sample includes outcome means for county-by-year observations. C&S=Callaway & Sant’Anna (2021)

**Appendix Figure 1. C&S Difference-in-Differences Event-Study Estimates of Obstetric Unit Closure Effects on Math and Reading Scores Allowing for Closure Effect One Year before Closure (i.e. Two Years before Closure as Reference Year)**


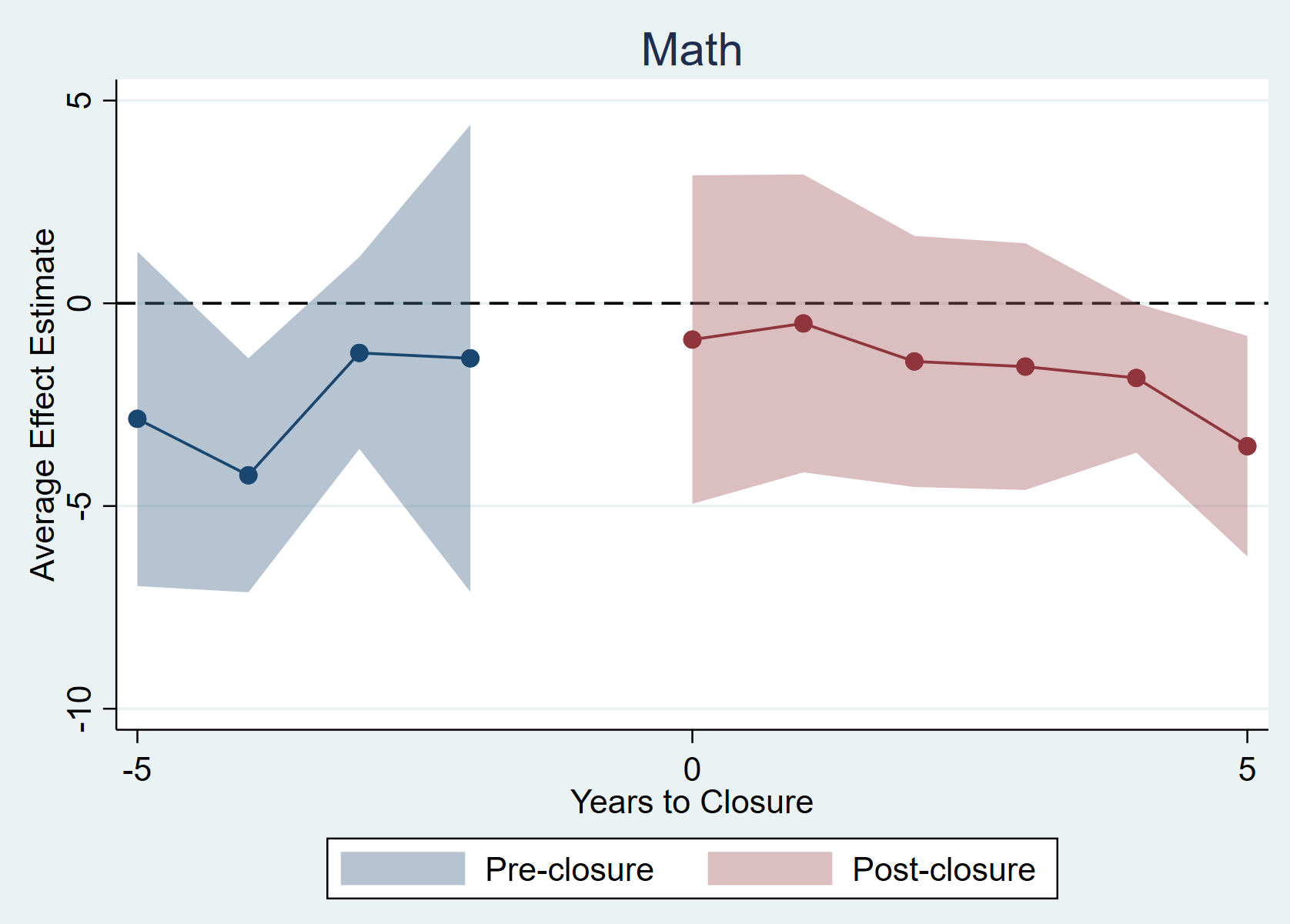

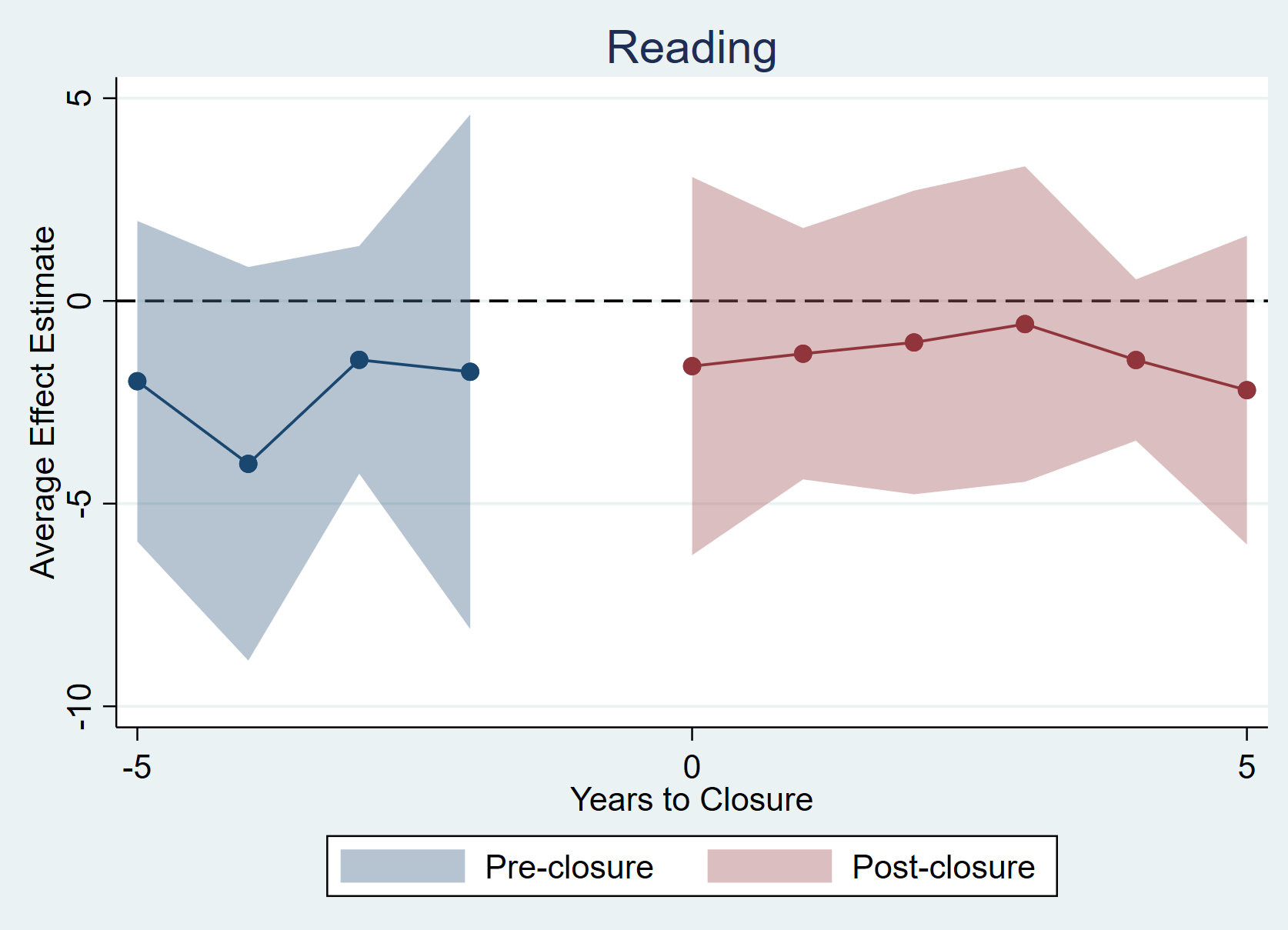


Notes: The dots represent the difference-in-difference event study estimates. The shaded area represents 95% confidence intervals for the estimates. Analytical sample includes outcome means for county-by-year observations. C&S=Callaway & Sant’Anna (2021).

**Appendix Figure 2. C&S Difference-in-Differences Event-Study Estimates of Obstetric Unit Closure Effects on Infant Outcomes Allowing for Closure Effect One Year before Closure (i.e. Two Years before Closure as Reference Year)**


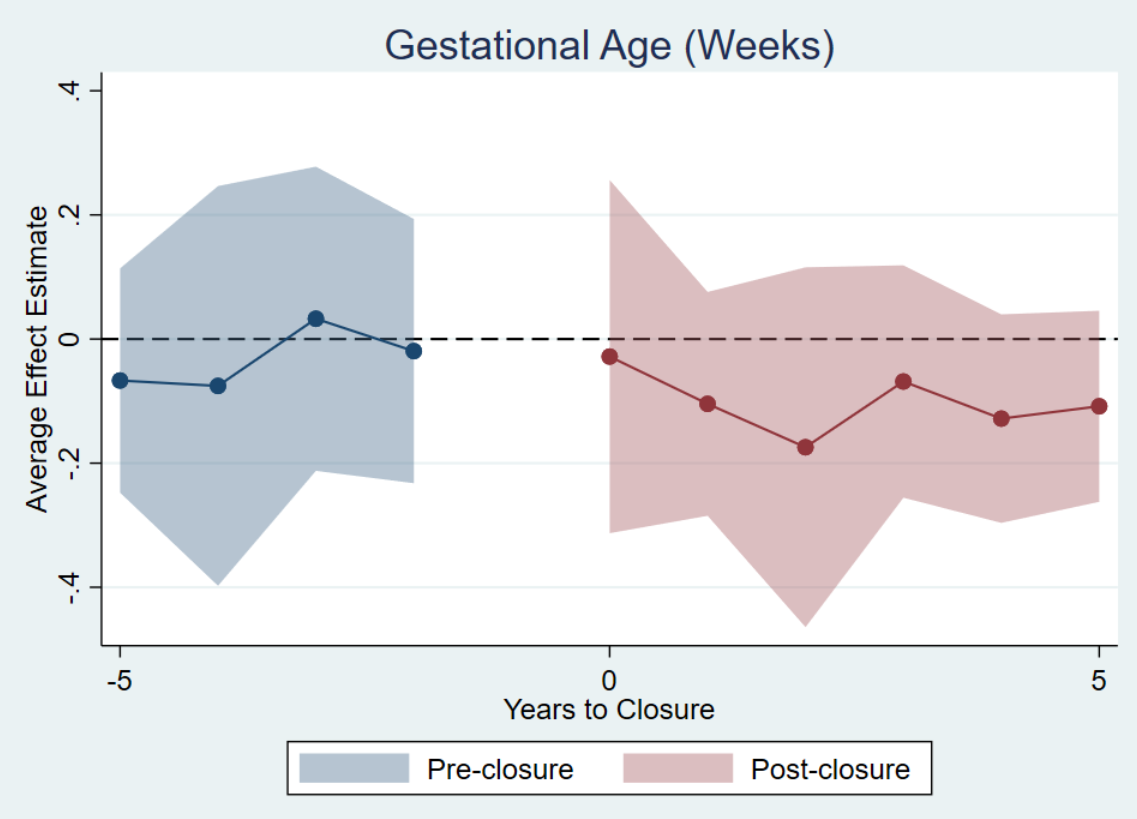

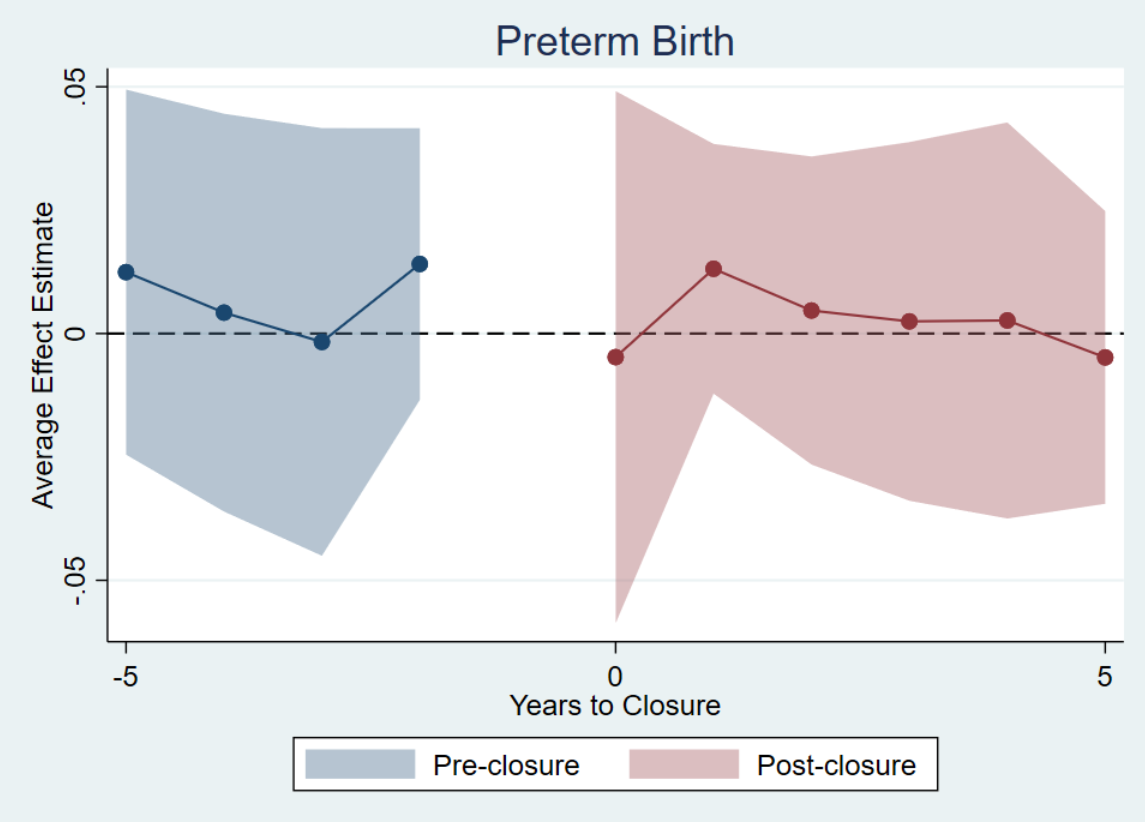


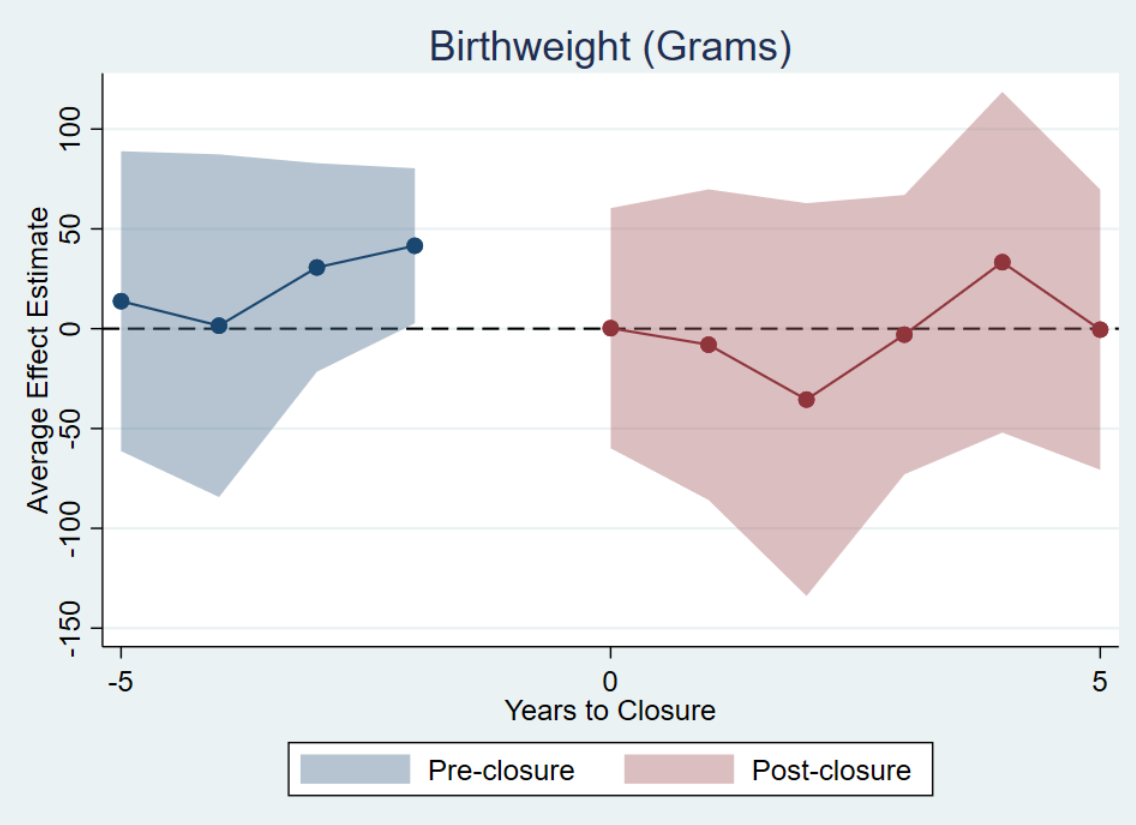

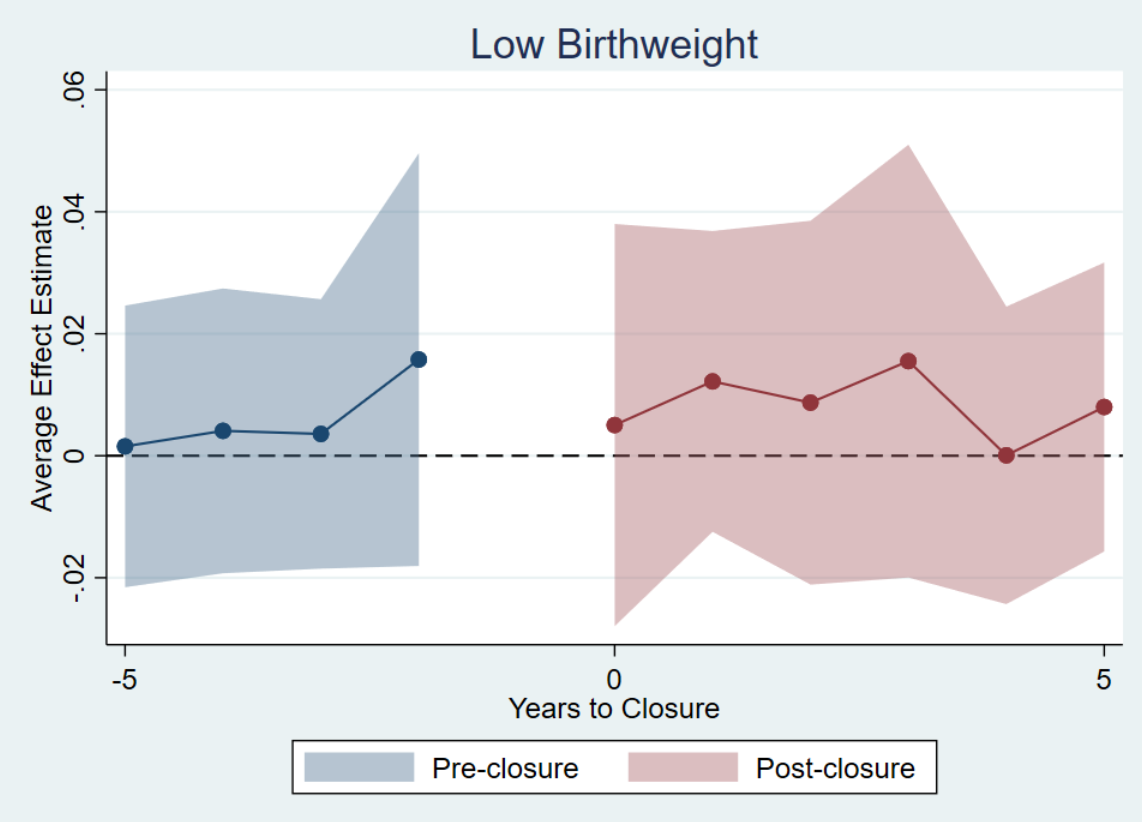


Notes: The dots represent the difference-in-difference event study estimates. The shaded area represents 95% confidence intervals for the estimates. The point estimates are in weeks for gestational age, grams for birth weight, and 0-1 likelihood change for preterm birth and low birthweight. Analytical sample includes outcome means for county-by-year observations. C&S=Callaway & Sant’Anna (2021).

**Appendix Figure 3. C&S Difference-in-Differences Event-Study Estimates of Obstetric Unit Closure Effects on Maternal Outcomes Allowing for Closure Effect One Year before Closure (i.e. Two Years before Closure as Reference Year)**


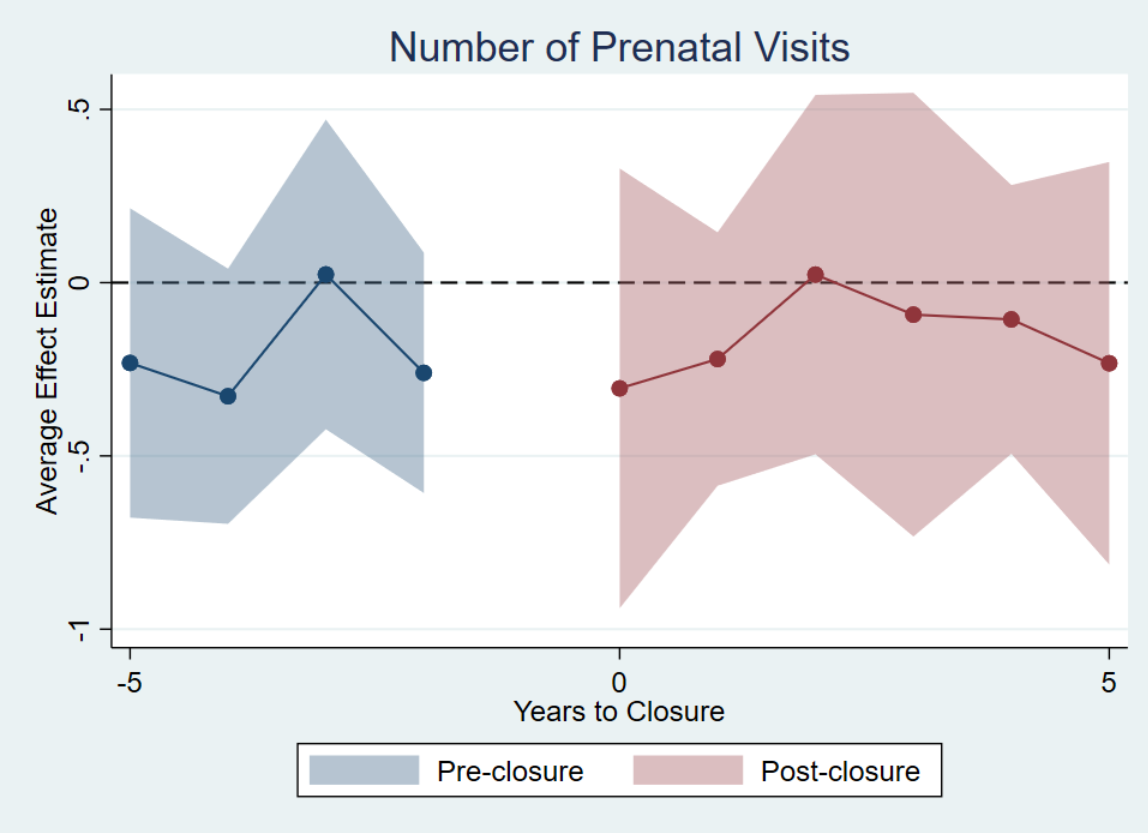

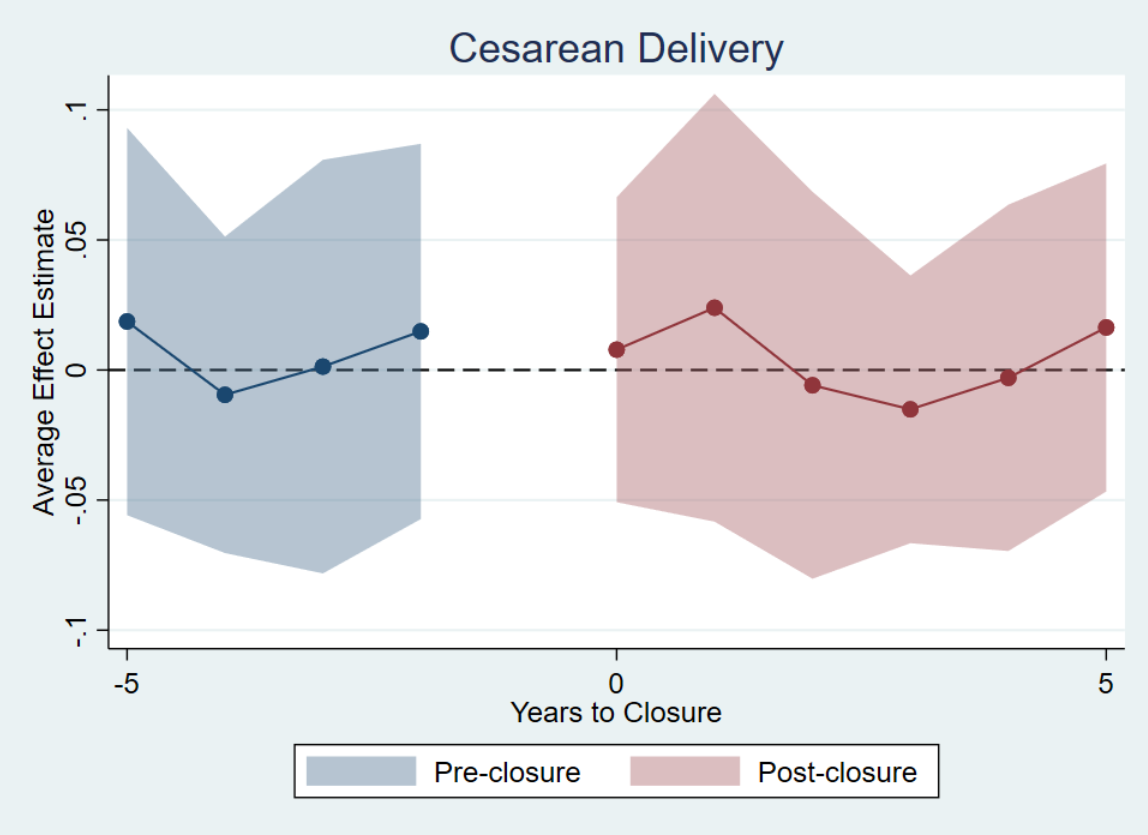

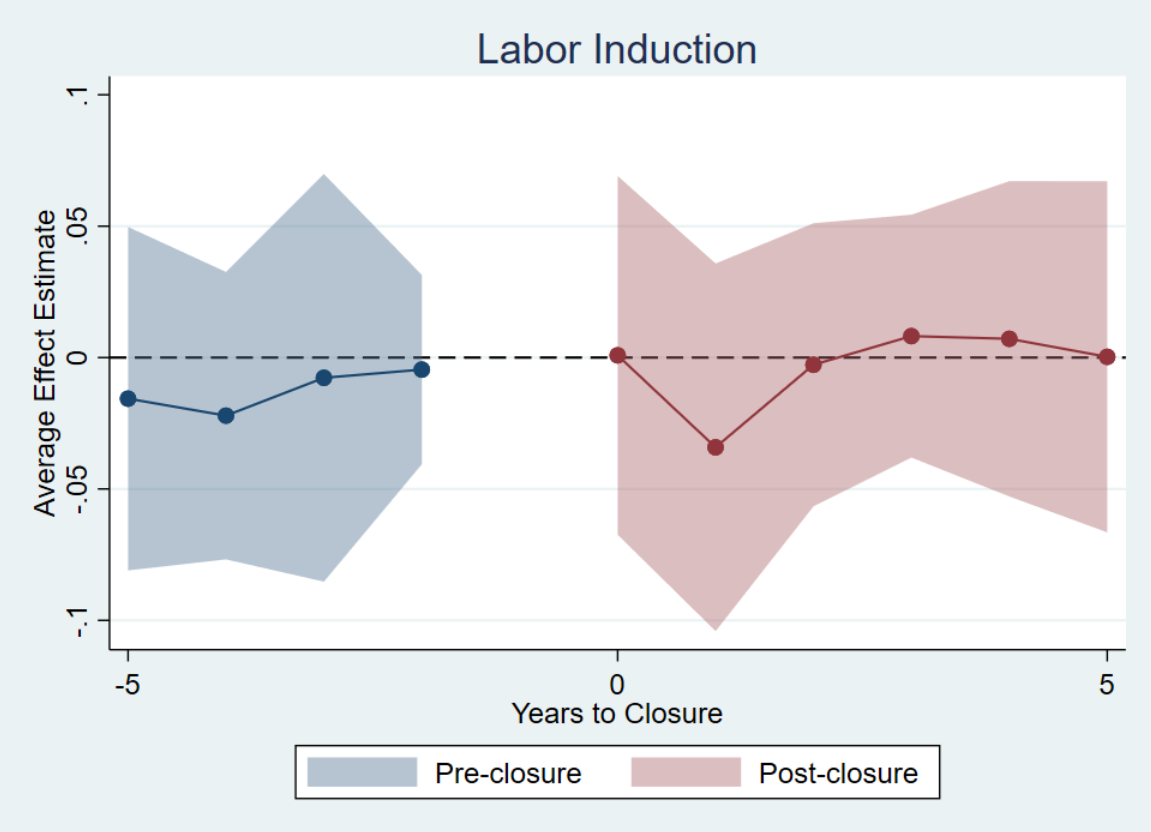


Notes: The dots represent the difference-in-difference event study estimates. The shaded area represents 95% confidence intervals for the estimates. The point estimates are in 0-1 likelihood changes for the outcomes. Analytical sample includes outcome means for county-by-year observations. C&S=Callaway & Sant’Anna (2021).
